# Low adherent cancer cell subpopulations are enriched in tumorigenic and metastatic epithelial-to-mesenchymal transition-induced cancer stem-like cells

**DOI:** 10.1038/srep18772

**Published:** 2016-01-11

**Authors:** Cynthia Morata-Tarifa, Gema Jiménez, María A. García, José M. Entrena, Carmen Griñán-Lisón, Margarita Aguilera, Manuel Picon-Ruiz, Juan A. Marchal

**Affiliations:** 1 Biopathology and Medicine Regenerative Institute (IBIMER), University of Granada, Granada, Spain; 2 Biosanitary Institute of Granada (ibs.GRANADA), University Hospitals of Granada-Univesity of Granada, Granada, Spain; 3 Department of Human Anatomy and Embryology, University of Granada, Granada, Spain; 4 Department of Oncology, University Hospital Virgen de las Nieves, Granada, Spain; 5 Institute of Neuroscience, Biomedical Research Center, University of Granada, Granada, Spain; 6 Animal Behavior Research Unit, Scientific Instrumentation Center, University of Granada, Granada, Spain; 7 Department of Microbiology, University of Granada, Granada, Spain; 8 Braman Family Breast Cancer Institute, Sylvester Comprehensive Cancer Center, University of Miami, Miller School of Medicine, Miami, Florida, USA

## Abstract

Cancer stem cells are responsible for tumor progression, metastasis, therapy resistance and cancer recurrence, doing their identification and isolation of special relevance. Here we show that low adherent breast and colon cancer cells subpopulations have stem-like properties. Our results demonstrate that trypsin-sensitive (TS) breast and colon cancer cells subpopulations show increased ALDH activity, higher ability to exclude Hoechst 33342, enlarged proportion of cells with a cancer stem-like cell phenotype and are enriched in sphere- and colony-forming cells *in vitro*. Further studies in MDA-MB-231 breast cancer cells reveal that TS subpopulation expresses higher levels of *SLUG*, *SNAIL*, *VIMENTIN* and *N-CADHERIN* while show a lack of expression of *E-CADHERIN* and *CLAUDIN*, being this profile characteristic of the epithelial-to-mesenchymal transition (EMT). The TS subpopulation shows *CXCL10*, *BMI-1* and *OCT4* upregulation, differing also in the expression of several miRNAs involved in EMT and/or cell self-renewal such as miR-34a-5p, miR-34c-5p, miR-21-5p, miR-93-5p and miR-100-5p. Furthermore, *in vivo* studies in immunocompromised mice demonstrate that MDA-MB-231 TS cells form more and bigger xenograft tumors with shorter latency and have higher metastatic potential. In conclusion, this work presents a new, non-aggressive, easy, inexpensive and reproducible methodology to isolate prospectively cancer stem-like cells for subsequent biological and preclinical studies.

The cancer stem cell (CSC) model posits that tumors are maintained by a subpopulation of cells that self-renew and yield heterogeneous progeny with reduced proliferative potential^1^,^2^. Different molecular mechanisms activated in normal stem cells are also involved in CSC self-renewal, including the expression of certain embryonic stem cells-transcription factors (ES-TFs)^3^ or the similar regulation of several signaling pathways^4^,^5^. Additionally, short non-coding miRNAs are also able to modulate gene expression programs to maintain self-renewal in normal and CSCs^6^.

It is important to note that CSCs also underlie drug resistance, tumor recurrence and metastasis^1^,^2^,^7^. Drug resistance and tumor recurrence showed by CSCs are mainly explained by the overexpression of multidrug resistance (MDR) membrane proteins and the enzyme aldehyde dehydrogenase (ALDH), or their ability to maintain a quiescent state^8^. On the other hand, metastasis is one of the most crucial steps in cancer progression and the main cause of mortality^1^. During local invasion and distant metastasis, the associated cancer cells typically develop alterations in their shape as well as in their attachment to other cells and to the extracellular matrix^9^. Metastatic cancer cells are characterized for suffering an epithelial-to-mesenchymal transition (EMT), a process by which cancer cells lose their attachment to the epithelial niche and acquire a mesenchymal phenotype^10^. These cells then are transported through the vasculature and are disseminated to anatomically distant organ sites where are able to establish new neoplastic growths^11^,^12^.

The relevance of this cancer cell subpopulation has yield to develop methodologies for their identification and isolation. Breast CSCs are characterized by the phenotype CD44^+^/CD24^low/−^ 13,14, while the expression of the cell surface proteins CD133, CD44 and/or CD326 is related with colon CSCs properties^15^,^16^,^17^. Other CSCs characteristics that have been extensively used for their identification and isolation are their high ALDH activity and ability to exclude Hoechst 33342, which is used to determine the side population (SP) phenotype^1^,^18^,^19^,^20^,^21^. However, the isolation of CSCs based on all of these properties requires the fluorescence-activated cell sorting (FACS) method, an expensive and aggressive technique.

As it has been reported previously, cancer cells that undergo EMT acquire characteristics of CSCs^22^,^23^. On the other hand, it is known that during EMT process cells lose their adhesion ability with adjacent epithelial cells^9^. This property has been extensively used to remove mesenchymal cells from primary epithelial cell cultures following the protocol known as “differential trypsinization” developed by Owens *et al.* in 1974^24^. Here we report that the application of this technique in cancer cell cultures shows that cells selected by differential trypsinization differ in phenotypical and functional CSCs properties, including ALDH activity, SP proportion, xenograft tumor formation ability and metastatic potential, among others. As expected, trypsin-sensitive (TS) cancer cells subpopulations show increased CSCs properties when compared with the total population (TP) and/or the trypsin-resistant (TR) subpopulation.

## Materials and Methods

### Cell lines and cell culture

Breast (MCF7 and MDA-MB-231) and colon (HT-29 and T84) cancer cell lines were obtained from American Type Culture Collection (ATCC) and cultured following ATCC recommendations.

### Differential trypsinization

**Figure 1:**
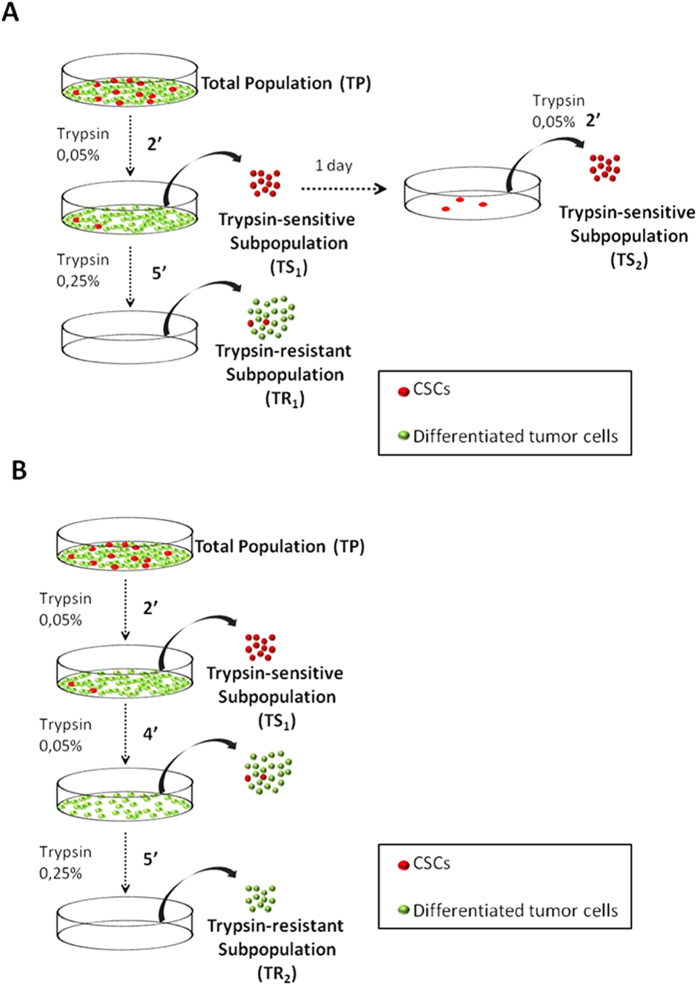
Differential trypsinization protocols. **(A)** Isolation of TS_1_, TS_2_ and TR_1_ subpopulations, used for Aldefluor assays, Hoechst 33342, surface markers, spheres and colonies formation ability. **(B)** Improved protocol to isolate highly attached cells (TR_2_) used for Aldefluor, qRT-PCR and *in vivo* assays. Red balls represent cells with higher CSC-like properties and green balls cells with a non CSC-like phenotype.

Cells at 60–80% of confluence were slowly washed with phosphate buffered saline (PBS) without directly flow falls on the cells. Then, 0.05% trypsin was added and incubated 2 minutes at 37 °C. Detached cells were collected in centrifuge tubes and were named as Trypsin-Sensitive 1 (TS_1_). The remaining attached cells were washed twice and, then, 0.25% trypsin was added. These cells were named as Trypsin-Resistant 1 (TR_1_). On the other hand, TS_1_ cells were again plated for 24 hours. After that, 0.05% trypsin was added and incubated for 2 minutes at 37 °C. These detached cells were named as Trypsin-Sensitive 2 (TS_2_) (Fig. 1A).

In order to separate more strictly cells with stem-like and no stem-like properties, cells were grown until 60–80% of confluence and washed slowly with PBS. After obtaining the TS_1_ subpopulation, remaining cells attached to the dishes were washed twice with PBS and incubated with 0.05% trypsin for 4 minutes at 37 °C. Cells detached from this trypsinization were discarded. Dishes with remaining trypsin-resistant cells were named as TR_2_ (Fig. 1B). Total population (TP) was used as control cells.

### Flow cytometry analyses

ALDEFLUOR assays (Stem Cell Technologies) to detect ALDH1 activity were performed according to manufacturer’s instructions. Diethylaminobenzaldehyde (DEAB) was used as an ALDH1 inhibitor to set ALDH1 gates. Cell surface levels were determined with anti-human antibodies CD44-PE, CD24-APC, CD133-APC, CD326-FITC, CK18-FITC and CK20-FITC (Miltenyi Biotec). All samples were analyzed on a FACS CANTO II (BD Biosciences) using the FACS DIVA software.

### Side population assays

Hoechst 33342 exclusion (Side Population) assays were carried out as previously described^21^,^25^.

### Sphere-forming assay

3 × 10^3^ cells were washed with PBS and resuspended in spheres culture medium (DMEM:F12, 1% penicilin/streptomycin, B27, 10 μg/mL ITS, 1 μg/mL Hydrocortisone, 4 ng/mL Heparin, 10 ng/mL EGF, 20 ng/mL FGF) in ultra-low adherence 24-well plates (Corning). Spheres >75 μM diameter were counted after 6 days by light microscopy.

For the secondary sphere-forming assay, cells from primary spheres were collected by centrifugation, then dissociated with trypsin-EDTA and mechanically disrupted with a pipette. 10^3^ single cells were plated and resuspended in spheres culture medium in ultra-low adherence 24-well plates. Spheres >75 μM diameter were counted after 6 days by light microscopy.

### Soft agar assay

10^4^ cells were seeded in 0.4% cell agar base layer, which was on top of 0.8% base agar layer in 6-well culture plates. Cells were then incubated for a further 21 days at 37 °C and 5% CO_2_. Cell colony formation was then examined under a light microscope after staining with 0.01% Crystal Violet (Sigma-Aldrich).

### qRT-PCR

Total RNA extraction and qRT-PCR assays were done following standard protocols. See Supplementary Material and Methods for more details. Primer sequences are listed in Supplementary Table 1.

### Proliferation assay

Different subpopulations (TP, TS_1_ and TR_2_) were seeded in 24-well plates in medium supplemented with 10% FBS for 6 days. Cells were incubated with MTT every two days and the fluorescence was measured at 570 nm.

### Lentivirus transfections

293T cells were co-transfected with a lentiviral vector encoding for the firefly luciferase and the red fluorescent protein td-Tomato (L2T)^26^, a packaging vector (psPAX2) and an envelope vector (pCMV-VSVG) using lipofectamine transfection reagent (Life Technologies). Viral particles produced from 293T cells were collected and used to infect MDA-MB-231 cells. Briefly, supernatant recovered after 48h from 293T transfected cells was filtered by a 0.45 μm pore membrane and added to MDA-MB-231 plated cells supplemented with 4 μg/mL polybrene (Sigma-Aldrich). After viral infection, td-Tomato^+^ cells were selected for stable integration of the transfects by FACS.

### Mouse models

Tumor-initiation experiments and experimental lung metastasis assays are detailed in Supplementary Materials and Methods. All animal studies were performed according to protocols reviewed and approved by the Institutional Animal Care and Use Committee at the University of Granada.

### Histological and immunological analysis

Described in detail in Supplementary Materials and Methods.

### Statistical analysis

All graphed data are presented as mean ± SD from at least three experiments. two-tailed Student’s t-tests and two-way ANOVA were used to determine statistical differences for *in vitro* and *in vivo* experiments respectively. P-values <0.05 were considered statistically significant in all cases.

## Results

### Trypsin-sensitive subpopulation displays CSC-like phenotypic properties

**Figure 2:**
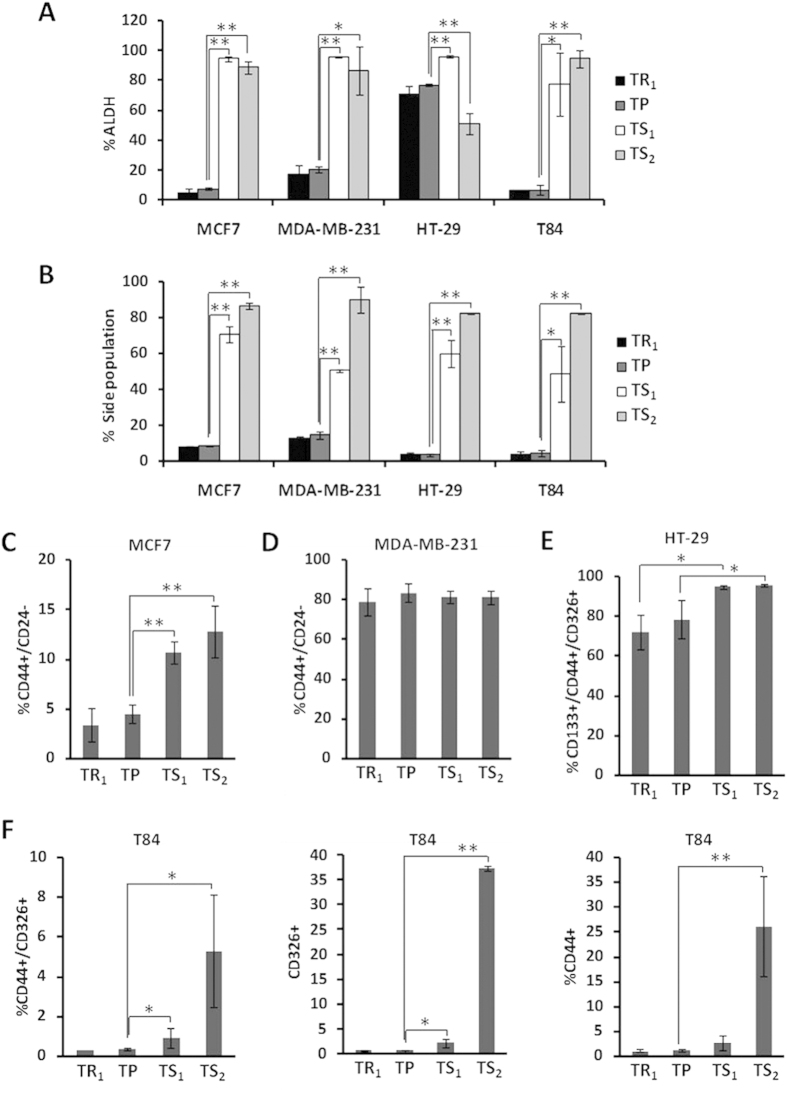
Phenotypic properties of breast and colon cancer cell subpopulations isolated by differential tripsinization. Percentage of ALDH^+^ **(A)** and SP **(B)** cells determined in subpopulations of MCF7, MDA-MB-231, HT-29 and T84 cancer cell lines. **(C,D)** Percentage of CD44^+^/CD24^−^ cells in TR_1_, TP, TS_1_ and TS_2_ isolated from MCF7 **(C)** and MDA-MB-231 **(D)** breast cancer cells measured by flow cytometry. **(E)** Co-expression of CD44, CD133 and CD326 in HT-29 cells isolated by differential trypsinization and in the TP. **(F)** Flow cytometry for CD44 and CD326 expression, alone or together, in T84 cells. Data are graphed as mean ± SD from at least two different experiments carried-out by triplicates (**P < 0.01; *P < 0.05). See also Supplementary Fig. S1.

To test whether TS subpopulations are enriched in CSC-like properties, we determined the ALDH activity, the rate of SP and specific breast and colon CSC-related cell surface markers (Fig. 2). Regarding to ALDH activity, both TS_1_ and TS_2_ subpopulations presented a significantly higher proportion of ALDH positive cells than TP. In contrast, TR_1_ subpopulation displayed a lesser ALDH activity, although this change was not significant when compared to the TP (Fig. 2A, Supplementary Fig. S1A and Supplementary Table 2).

Accordingly, the rate of SP in TS subpopulations was significantly higher than in both TP and TR_1_ cells for all cell lines tested. Thus, TS_1_ cells showed values ranged from 48.6% up to 70.5% and TS_2_ from 82.3% up to 89.9%. However, TP cells displayed percentages comprising between 3.9% and 13.2% (Fig. 2B, Supplementary Fig. S1B and Supplementary Table 3).

Moreover, we determined CD44/CD24 expression for breast and CD44/CD133/CD326 expression for colon cancer cell lines to study the proportion of cancer cells with a CSC phenotype in the different subpopulations. Flow cytometry analysis indicated that in the MCF7 cell line, TS_1_ and TS_2_ subpopulations showed a significantly higher CD44^+^/CD24^−^ proportion than TP and TR_1_ (Fig. 2C). However, no significant differences were found for these markers in the different subpopulations of the MDA-MB-231 breast cancer cell line, probably due to the high proportion of cells with this phenotype in the TP (Fig. 2D). In HT-29 colon cancer cells, the expression of CD44^+^/CD133^+^/CD326^+^ was significantly higher for TS_1_ and TS_2_ in comparison to TR_1_, although only TS_2_ showed a significant increase in the proportion of cells with this phenotype when compared to the TP (Fig. 2E). It is important to note that HT-29 cells showed two different subpopulations based on CD326 expression, and that CD326^high^ and CD326^low^ HT-29 cells were separated prospectively by differential trypsinization, being the TS subpopulation composed for CD326^high^ cells (Supplementary Fig. S1C). In regards to T84 colon cancer cells, TS_1_ and TS_2_ showed a higher enrichment in cells with a CD44^+^/CD326^+^ phenotype, even when their expression was studied together or separate (Fig. 2F). Differences in the expression of CD133 were not significant; however, this cell surface marker was more expressed in TS subpopulations (Supplementary Fig. S1D).

### Trypsin-sensitive subpopulation possesses higher self-renewal ability and clonogenicity

**Figure 3:**
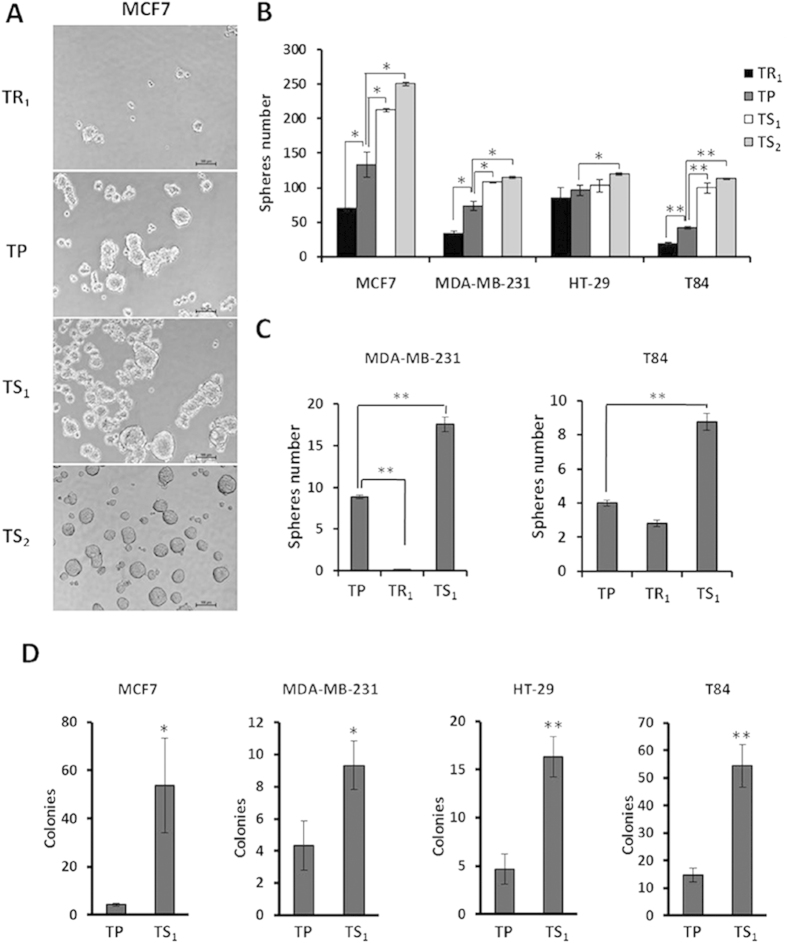
Tumorsphere- and colony-forming ability of breast and colon cancer cell subpopulations isolated by differential trypsinization. (**A**) Representative images of mammospheres formed from different MCF7 subpopulations. (**B**) Number of spheres formed by different subpopulations of each cell line. (**C**) Secondary sphere formation assay performed in MDA-MB-231 and T84 cells selected by differential trypsinization and compared to the TP. Data shown as mean ± SD (**P < 0.01; *P < 0.05). (**D**) Number of colonies formed by TP and TS1 of each cell line. Data are represented as mean ± SD (**P < 0.01; *P < 0.05).

We next tested *in vitro* functional characteristics of these cell subpopulations isolated by differential trypsinization. We studied the anchorage-independent growth of TS and TR subpopulations under free-serum conditions to determine their self-renewal ability; and also their clonogenic capacity by colony-formation assay in soft agar. As it is shown in Fig. 3A,B, tumorspheres were formed more efficiently from TS_1_ and TS_2_ than TR_1_ and TP cells in all cell lines; therefore TS subpopulations have a higher capacity of self-renewal. Moreover, TR_1_ subpopulation formed significantly less spheres than TP for MCF7, MDA-MB-231 and T84 (Fig. 3A,B).

We also performed a secondary sphere-formation assay by dissociating primary spheres formed by TS_1_, TR_1_ and TP cells, as indicated in the Materials and Methods section. As it is shown in Fig. 3C for the breast cancer MDA-MB-231 and colon cancer T84 cell lines, the number of cells with the ability to form secondary spheres was higher in primary spheres formed by the TS_1_ subpopulation, while those formed by TR_1_ were poorly enriched in sphere-forming cells. In addition, TS subpopulations showed a significant higher capacity to form colonies in soft agar than TP for all cell lines studied (Fig. 3D). These data are in concordance with our previous results obtained from the primary sphere formation assay and support our prior conclusion about the higher self-renewal ability of the TS subpopulation.

All together, these results indicate that cells isolated by differential trypsinization differ in their enrichment in cells with functional CSC-like properties. Furthermore, the rate of these cells in the subpopulations isolated is well maintained when cells are cultured in suspension in FBS-free media.

### Isolation of cancer cells with stem- and no stem-like properties based on their adhesion capacity

**Figure 4:**
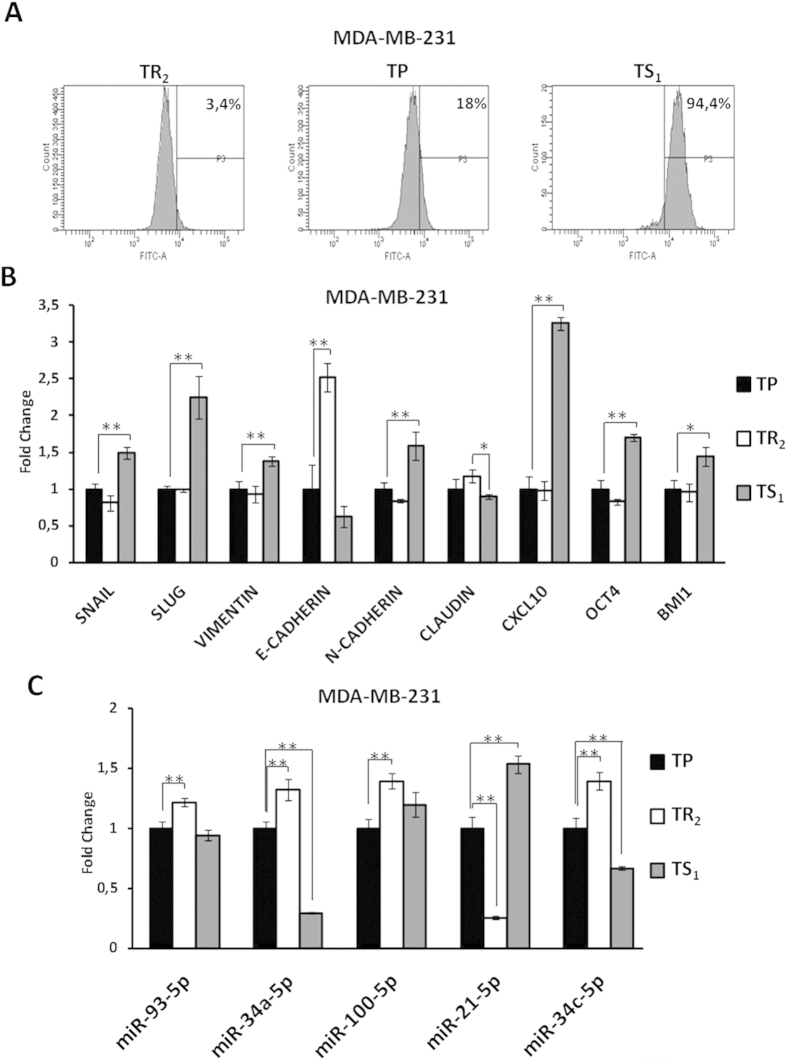
ALDH activity, mRNA and miRNA expression in enriched subpopulations of MDA-MB-231 cells isolated by their different adhesion capacity. (**A**) Aldefluor assay performed in TR_2_, TP and TS_1_ MDA-MB-231 cells. See also Supplementary Fig. S2. (**B**) qRT-PCR analysis for the expression of CSCs and EMT-related genes in different subpopulations of MDA-MB-231 cells. Data are normalized to 1 for TP using *GAPDH* as internal control, and graphed as mean ± SEM (n = 3) (**P < 0,01; *P < 0,05). (**C**) Differential expression of miRNAs related to CSC phenotype and/or EMT in MDA-MB-231 cells isolated by differential trypsinization. Data are normalized to 1 for TP using miR-24c-3p as internal control, and graphed as mean ± SEM (n = 3) (**P < 0.01; *P < 0.05).

As we shown above, we obtained a cell subpopulation enriched for CSCs features based in their lower adhesion capacity to the cell culture surface. The amount of cells detached after the first trypsinization was very low and the rest of attached cells was very similar to the total population. For this reason, to demonstrate that it is possible to separate CSCs and cancer cells no stem based on their sensitivity to trypsin digestion, we developed another protocol. After the first trypsinization we recovered the TS_1_ subpopulation and remaining attached cells were trypsinized again with the same trypsin dilution indicated above, but in this case for 4 minutes. Cells that were detached by this last trypsinization were discarded and cells still attached were recovered and named as TR_2_, as described in the Material and Methods section (Fig. 1B). As it is shown in Fig. 4A and Supplementary Fig. S2, TP and subpopulations TS_1_ and TR_2_ differed in ALDH activity. In concrete, TS_1_ subpopulation was enriched in cells with a high ALDH activity, with purity higher than 90%, and TR_2_ showed a lack of ALDH activity in both MDA-MB-231 and MCF7 (Fig. 4A and Supplementary Fig. S2). As it is shown the purity of ALDH^+^ cells obtained by differential trypsinization from MCF7 cells was similar to the enrichment obtained by FACS (Supplementary Fig. S2).

On the other hand, we also studied the expression levels of both the luminal cytokeratin 18 and the intestinal epithelium differentiation cytokeratin 20 for breast and colon cancer cells, respectively, after differentiation of TS_1_ cells in media containing FBS for 7 days when grown attached to the cell culture surface. As it is shown in Supplementary Fig. S3, TS_1_ cells cultured under these conditions recovered the expression levels of these cytokeratins at similar values than those observed in the TP. These results are an indicator of the ability of the TS_1_ cells to recapitulate the heterogeneity of the parental cell line.

### Trypsin-sensitive subpopulation expresses higher levels of EMT- and pluripotency- associated genes

The enrichment of cancer cells with stem cell-like properties in cells which show low adherence to plastic surface may be due to an EMT process, which is associated with metastasis. To understand the molecular changes underlying the different characteristics of these cell subpopulations, we analyzed the expression of genes related with the EMT process and/or pluripotency on MDA-MB-231 cells.

qRT-PCR studies demonstrated that all genes assayed were expressed differentially in the TS_1_ and the TR_2_ cell subpopulations. *SNAIL*, *SLUG*, *VIMENTIN*, *N-CADHERIN*, *CXCL10*, *OCT4* and *BMI1* were overexpressed in the TS_1_ subpopulation when compared to the TP, and underexpressed in the TR_2_ subpopulation (Fig. 4B). In contrast, expression levels of *CLAUDIN1* and *E-CADHERIN* were higher in TR_2_ cells and much lower in the TS_1_ subpopulation (Fig. 4B).

On the other hand, the expression patterns of several miRNAs related with EMT and/or pluripotency also differ between these two cell subpopulations isolated by differential trypsinization. The TR_2_ subpopulation showed a significant increase in the expression levels of miR-93-5p, miR-34a-5p, miR-100-5p and miR-34c-5p (Fig. 4C). However, all of these miRNAs were downregulated in the TS_1_ subpopulation when compared to the TP, although not all of them were statistically significant (Fig. 4C). In contrast, the expression of miR-21-5p was higher in TS_1_ and lower in TR_2_ cells when compared to the TP (Fig. 4C).

**Figure 5:**
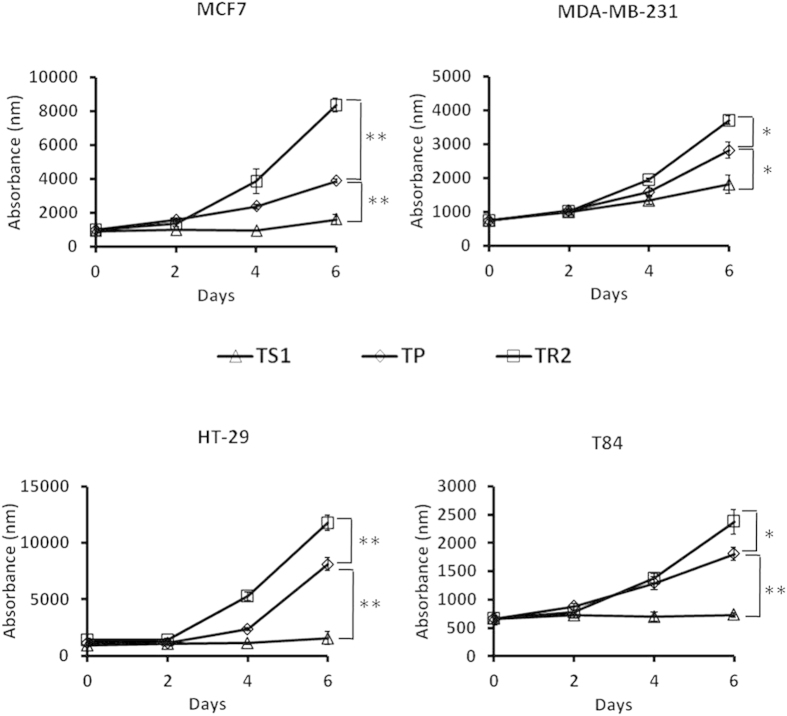
Proliferation assay. Proliferation curves of TS_1_, TR_2_ and TP cells cultured for 6 days in media containing FBS, and measured using MTT every 2 days in cultures seeded with an equal number of cells at day 0. Data are represented as absorbance at 570 nm and shown as mean ± SD (**P < 0.01; *P < 0.05).

Moreover, the proliferation assay demonstrated that both TR_2_ and TP subpopulations grow faster that TS_1_ for all tumor cell lines (Fig. 5), showing that the TS_1_ subpopulation has a lower proliferation rate *in vitro*.

### Trypsin-sensitive subpopulation is enriched in tumor-initiating cells

**Figure 6:**
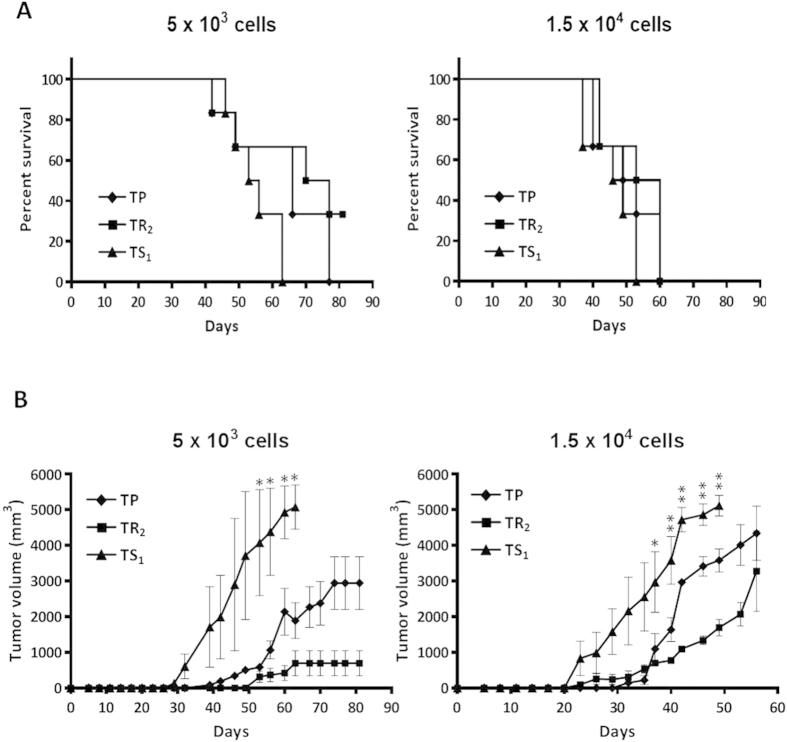
Overall survival and subcutaneous xenograft tumor formation **(A)** Kaplan-Meier curve for overall survival of NSG mice injected with 5 × 10^3^ cells (left) and 1.5 × 104 cells (right) from TS1, TR2 or TP. **(B)** Tumor volume (mm^3^) of subcutaneous xenograft tumors formed by 5 × 10^3^ and 1.5 × 10^4^ cells from TS_1_, TR_2_ and TP in NSG mice. Data are shown as mean ± SD (**P < 0.01; *P < 0.05).

To confirm the results observed *in vitro*, TS_1_ and TR_2_ subpopulations were isolated from MDA-MB-231 cells, injected subcutaneously in NSG mice and compared with the TP in a limiting dilution *in vivo* assay. The overall survival of mice injected with TS_1_ cells was lower than for the other groups (Fig. 6A). When 5 × 10^3^ TS_1_, TP or TR_2_ cells were injected, the overall survival of mice was 63 and 77 days, for TS_1_ and TP respectively, while 2 out of 6 mice injected with the TR_2_ subpopulation were still alive at day 81, the end-point of the experiment (Fig. 6A). Furthermore, tumor volume was higher after injection of TS_1_ cells, while tumors formed by TR_2_ cells were smaller (Fig. 6B), being these results not explained by a global mitogenic effect since TR_2_ cells have higher proliferation rate than TS_1_ cells (Fig. 5). Similar results were observed when 1.5 × 10^4^ cells were injected, however the overall survival was shorter and the tumor size bigger for all groups, as it was expected (Fig. 6A,B). All together, these data demonstrate that TS cells are enriched in CSCs with a high tumor-initiating capacity while the TR subpopulation is composed mainly by non CSC-like cells.

### Trypsin-sensitive subpopulation generates more metastasis than trypsin-resistant subpopulation after tail vein injection

**Figure 7:**
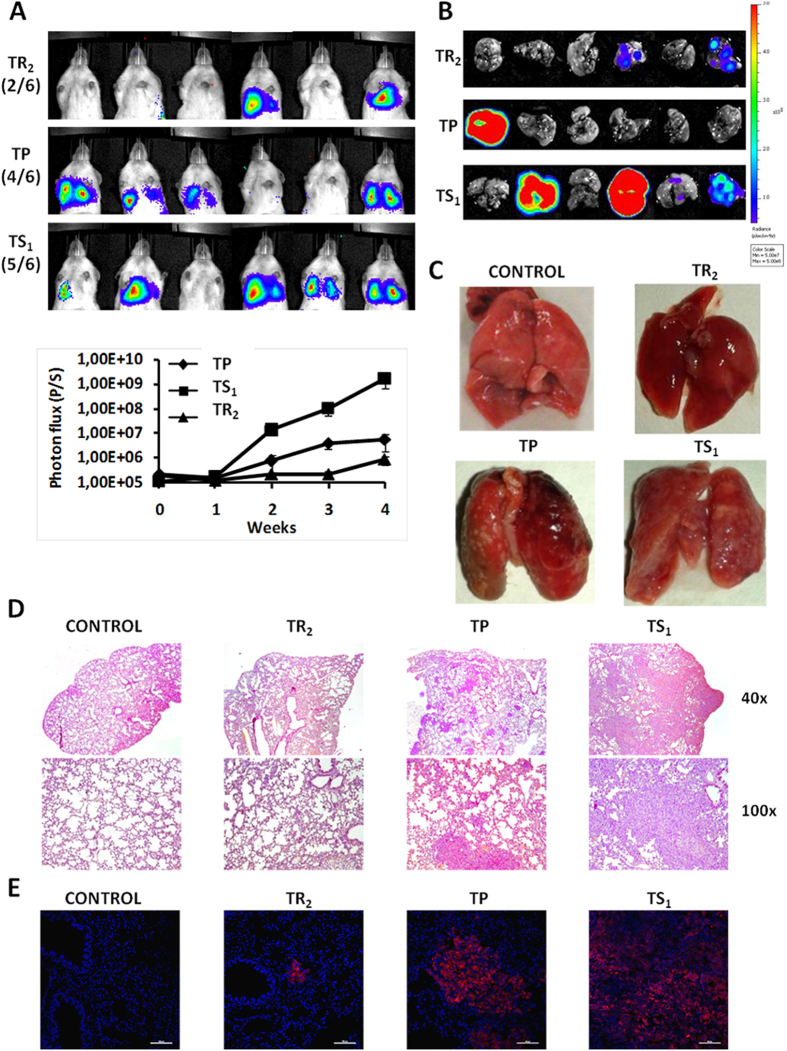
Experimental lung metastasis assay. **(A)** IVIS images of lung metastasis formed after tail vein injection of TP, TR_2_ and TS_1_ MDA-MB-231 L2T cells (up). Measure of photon flux is graphed as mean ± SEM (down). **(B)** Fluorescence of metastatic lungs *ex vivo*. **(C)** Representative optical images of lung metastasis for a healthy mice (control) and lungs extracted from NSG mice injected with different subpopulations of MDA-MB-231 cells obtained by differential trypsinization. **(D,E)** Histological **(D)** and immunohistochemical **(E)** images of lungs obtained from healthy mice (control) and mice injected with TR_2_, TP and TS_1_ cells (left to right).

To further demonstrate the higher aggressiveness of the TS subpopulation, we compared the metastatic ability of both cell subpopulations isolated by differential trypsinization (TS_1_ and TR_2_) with the TP using MDA-MB-231 L2T cells. When 2.5 × 10^5^ TS_1_ cells were injected into the tail vein of NSG mice, 5 of 6 animals developed lung metastasis, however only 4 and 2 of 6 animals developed metastasis when TP or TR_2_ cells, respectively, were injected (IVIS images from week 3 are shown in Fig. 7A). In addition, the TS_1_ subpopulation, that showed the lowest proliferation rate *in vitro* (Fig. 5), caused a more rapid increase in tumor bioluminescence, demonstrating their higher metastatic ability (Fig. 7A). After 4 weeks, animals were sacrificed and lungs harvested for further analysis (Fig. 7B). Lungs from mice injected with TS_1_ cells showed more pulmonary metastatic nodules than these from TP or TR_2_ cells (representative images, Fig. 7C). Furthermore, histological and immunohistochemical analysis of lungs demonstrated that metastatic nodules formed by TS_1_ cells were bigger when compared to the TP, and smaller when TR_2_ cells were injected (Fig. 7D,E), being these differences in size independent of a global mitogenic effect (Fig. 5). This further demonstrates the higher metastatic ability of the TS subpopulation and that the TR subpopulation is composed by non CSC-like cells.

## Discussion

In this work, we demonstrate that cultured cancer cells possess low adherent cancer cell subpopulations with stemness properties. Therefore, differential trypsinization, an extended methodology for the establishment of primary epithelial cell cultures^24^,^27^, can be applied to isolate breast and colon cancer cells with CSCs properties *in vitro* and *in vivo*, and likewise for other cancer types with an epithelial origin. In this work, we demonstrate that two distinct subpopulations of cancer cells can be isolated by this protocol: i) a subpopulation with CSCs properties and lower adherence ability (TS subpopulation); ii) and a highly adherent subpopulation enriched in non CSC-like cells (TR subpopulations).

Breast and colon cancer TS subpopulation is enriched in cells with high ALDH activity, and greater SP rate, being these characteristics associated with CSCs properties. The isolation of a subpopulation with high ALDH activity has been demonstrated to show higher tumorigenic potential in both breast and colon cancers^18^,^20^. Regarding to Hoechst 33342 dye efflux, different studies have shown that the SP phenotype is enriched in cancer stem-like cells in different tumors, including breast and colon cancers^19^,^21^. Moreover, the ability to exclude Hoechst 33342 is associated with an upregulation of ATP-binding cassette transporters that confer multidrug resistance, a characteristic of CSCs^28^.

However, the characterization of CSCs by the expression of specific cell surface markers is the most extended method to identify and isolate this cancer cell subpopulation. We have demonstrated that MCF7 breast cancer cells from the TS subpopulation are enriched in cells with a CD44^+^/CD24^−^ phenotype, which is characteristic of breast CSCs^13^. On the other hand, HT-29 and T84 TS colon cancer cells are enriched in CD44^+^, CD133^+^ and/or CD326^+^ cells. Colon CSCs were firstly identified based on the expression of CD133, being determined that the ability to develop tumors in immunodeficient NOD/SCID mice was restricted to CD133^+^ cells^15^,^16^. In a latter study, Dalerba *et al.* demonstrated that the ability to engraft in immunodeficient mice was restricted to a minority subpopulation CD326^high^/CD44^+^. Furthermore, tumors originated from CD326^high^/CD44^+^ cells maintained a differentiated phenotype and reproduced the full morphologic and phenotypic heterogeneity of their parental lesions^17^. Regarding to MDA-MB-231 TS cells, we did not see a significant increase in the CD44^+^/CD24^−^ subpopulation, however the percentage in all groups was very high, around 80%, which is in concordance with the highly invasive/metastatic skills of this triple negative breast cancer cell line^29^.

Breast and colon cancer cells isolated by differential trypsinization also differ in their self-renewal ability. CSC self-renewal can be assayed *in vitro* by tumor spheres and holoclone colonies formation assays^30^. The TS subpopulation obtained from both breast and colon cancer cell lines generated more colonies in soft agar and spheres in low attachment plates under serum deprivation. It has been demonstrated that colon CSCs enriched in CD133^+^ cells form more colonies in soft agar used as indicator of their higher tumor-initiating ability^31^. In addition, it has been shown that MDA-MB-231 breast cancer cells enriched in cancer-initiating stem cells have higher colony-formation ability^32^. On the other hand, CSCs have the ability to form spheres in low-attachment culture surfaces under free serum conditions and this kind of culture conditions increases the percentage of cells with a CSC phenotype based on surface markers expression^25^,^33^. In contrast, the number of colonies and spheres formed from TR cells was significantly lower than in the TP, indicating the lower self-renewal ability and therefore, the non CSC-like properties of this cancer cell subpopulation selected by a higher adhesion capability to the cell culture surface.

All these *in vitro* studies for phenotypical and functional properties of CSCs were notably increased in the TS subpopulations for all cell lines, except for CD44^+^/CD24^−^ expression in MDA-MB-231 breast cancer cells that is basally high in TP cells. For this reason, we selected this cell line to perform further studies. qRT-PCR analysis of different mRNA and miRNA related with EMT, self-renewal and/or aggressiveness demonstrated differences in their expression between TR and TS subpopulations. The gene profile of MDA-MB-231 TS cells was more associated with a mesenchymal phenotype while the TR subpopulation was more related with an epithelial phenotype. During the EMT process, cancer cells lose their attachment to the epithelial niche and acquire a mesenchymal phenotype which is characteristic of metastatic cancer cells^34^. Moreover, the triple-negative MDA-MB 231 breast cancer cell line, which overexpresses EGFR^35^, has potent and reproducible migratory responses^36^. MDA-MB-231 TS cells showed a lack of expression of *E-CADHERIN* and *CLAUDIN*. The loss by carcinoma cells of E-cadherin, a key cell-to-cell adhesion molecule which establish adherent junctions with adjacent epithelial cells, is the best characterized alteration during the EMT process^9^,^34^. In regard to claudin expression, a phenotype claudin-low is associated with enrichment for EMT markers, immune response genes, cancer stem cell-like features and poor outcome in breast cancer^37^. In addition, the TS subpopulation also shows a higher expression of several transcription factors that modulates the EMT, such as *SNAIL* or *SLUG*, and other mesenchymal markers including *N-CADHERIN* and *VIMENTIN*^38^. In contrast, the expression of these genes was contrary regulated in MDA-MB-231 TR cells, indicating their more epithelial features. In fact, we have recently demonstrated important role of Vimentin/Slug EMT markers in tumor growth of primary breast cancer patients and in circulating tumor cells (CTCs) EGFR^+^ from cytokeratin negative non-metastatic breast cancer patients^39^.

Certain ES-TFs, such as Oct4, Sox2 and Nanog, which are master regulators of ES self-renewal and pluripotency, are also upregulated in CSCs^40^. *OCT4* expression was measured in MDA-MB-231 cells, demonstrating that it is overexpressed in the TS subpopulation. Interestingly, a recent study showed that Oct4 is associated with poor prognosis and that positively regulates the EMT process, contributing to breast cancer metastasis^38^. Furthermore, CSCs share similarities with normal stem cells in the regulation of the Wnt, Notch and Hedgehog signaling pathways, and the expression of several genes of the polycomb family, including *BMI-1*, or the *HOX* family among others which are involved in stem cells self-renewal^4^. *BMI-1*, which is overexpressed in TS cells, has been also related with the EMT process and drug resistance^41^. Moreover, *CXCL10* is expressed in basal tumors as compared with ER^+^ tumors, being associated with a higher tumor grade and a poor prognosis of breast cancer^42^.

On the other hand, miRNAs play critical regulatory roles in a wide range of biologic and pathologic processes, and miRNA expression profiling reveals substantial differences between cancer and normal tissue, and among different cancer types^43^. We studied the expression patterns of different miRNAs that have been previously shown to be related with CSCs properties, EMT, metastasis and poor outcome. For example, the TR subpopulation overexpresses miR-93, which inhibits cell proliferation, the capacity to form colonies^44^ and promotes mesenchymal-to-epithelial transition (MET)^45^. miR-34a-5p and miR-34c-5p are downregulated in TS cells and upregulated in the TR subpopulation. miR-34 family are reportedly as tumor-suppressor miRNAs implicated in reduced CSC properties and increased sensitivity to drug treatment by directly targeting *NOTCH1*^46^. miR-100 is also upregulated in the TR subpopulation, and its expression levels relate to the cellular differentiation state, with lowest expression in cells displaying stem cell markers^47^. In addition, the expression of miR-21, a well known miRNA which is dysregulated in several types of cancer and plays a key role in carcinogenesis, recurrence and metastasis^48^, is increased in the TS subpopulation and decreased in TR cells.

All together, these data demonstrate that differential trypsinization of breast and colon cancer cell cultures allows the isolation of two subpopulations (TR and TS) that differ in their CSC-like cells properties. TS cells show increased ALDH activity, SP proportion and CSC-like phenotype. This subpopulation is enriched in cells that have followed an EMT process and possess high self-renewal ability. Moreover, TS cells show higher tumorsphere- and colony-formation ability *in vitro*; and tumorigenic and metastatic potential *in vivo*. In contrast, the TR subpopulation is mainly composed by non CSC-like cells with a lower tumorigenic capacity both *in vitro* and *in vivo*. In a previous study carried-out by Walia and Elbe, it was shown that the TS subpopulation, obtained by differential trypsinization from transformed human mammary epithelial cells, was enriched in stem cell properties^49^. In a more recent study, this methodology was used to isolate a subpopulation of cells with a mesenchymal phenotype from an oncogenic transformed human mammary epithelial cell line^50^. However, these studies were limited to determine EMT markers expression, mammosphere-forming ability, drug resistance and CD44/CD24 expression, and were carried-out in a well differentiated oncogenic transformed human mammary epithelial cell line.

## Conclusion

In summary, our results evidence that cultured cancer cells possess low adherent cell subpopulations with stemness properties and with high tumorigenic and metastatic abilities. We show for the first time that differential trypsinization, a new, non-aggressive, easy, inexpensive and reproducible methodology to isolate prospectively breast and colon cancer stem-like cells for subsequent biological studies, high throughput screening therapeutic strategies targeting CSCs and preclinical assays.

## Additional Information

**How to cite this article**: Morata-Tarifa, C. *et al.* Low adherent cancer cell subpopulations are enriched in tumorigenic and metastatic epithelial-to-mesenchymal transition-induced cancer stem-like cells. *Sci. Rep.* **6**, 18772; doi: 10.1038/srep18772 (2016).

## Supplementary Material

Supplementary Information
