# Supplementary material for: Low adherent cancer cell subpopulations are enriched in tumorigenic and metastatic epithelial-to-mesenchymal transition-induced cancer stem-like cells
Source: Sci Rep. 2016 Jan 11;6:18772. doi: 10.1038/srep18772 (PMC4707518; doi:10.1038/srep18772)
Supplement: Supplementary Information [file srep18772-s1.pdf]

Low adherent cancer cell subpopulations are enriched in tumorigenic and metastatic epithelial-to-mesenchymal transition-induced cancer stem-like cells

Cynthia Morata-Tarifa[1,2], Gema Jiménez[1,2,3], María A. García[1,2,4], José M. Entrena[5,6], Carmen Griñán-Lisón[1,2], Margarita Aguilera[2,7], Manuel Picon-Ruiz[1,2,8], and Juan A. Marchal[1,2,3]

[1]Biopathology and Medicine Regenerative Institute (IBIMER), University of Granada, Granada, Spain. [2]Biosanitary Institute of Granada (ibs.GRANADA), University Hospitals of Granada-Univesity of Granada, Granada, Spain. [3]Department of Human Anatomy and Embryology, University of Granada, Granada, Spain. [4]Department of Oncology, University Hospital Virgen de las Nieves, Granada, Spain. [5]Institute of Neuroscience, Biomedical Research Center, University of Granada, Granada, Spain. [6]Animal Behavior Research Unit, Scientific Instrumentation Center, University of Granada, Granada, Spain. [7]Department of Microbiology, University of Granada, Granada, Spain. [8]Braman Family Breast Cancer Institute, Sylvester Comprehensive Cancer Center, University of Miami, Miller School of Medicine, Miami, Florida, USA.

Corresponding Authors: Juan A. Marchal, M.D., Ph.D, Department of Human Anatomy and Embryology, Faculty of Medicine, University of Granada, 18071 Granada, Spain, Ph: 958241000 Ext 20080, jmarchal@ugr.es; Manuel Picon-Ruiz, Ph.D, Sylvester Comprehensive Cancer Center, University of Miami Miller School of Medicine, Miami, FL 33136, USA, Ph: 3052437294, m.piconruiz@miami.edu

SUPPLEMENTARY DATA

MATERIALS AND METHODS

qRT-PCR

Total RNA from the different cell lines was extracted using the miRNeasy Mini Kit (Qiagen, Limburgo, Netherland) following the instructions of the manufacturer. Reverse transcription were realized using iScript^{TM} cDNA Synthesis Kit (Bio-Rad, Hercules, CA, USA) for mRNA and miRCURY LNA^{TM} Synthesis kit II (Exiqon, Vedbaek, Denmark) for miRNAs. qRT-PCR assay was done using iQ™ SYBR® Green Supermix (Bio-Rad) and miRCURY LNA^{TM} EXILENT SYBR Green (Exiqon) for miRNAs. Amplification of target cDNAs was performed using CFX96 real-time PCR detection (Bio-Rad). Each reaction was performed in triplicate. The comparative threshold cycle (Ct) method was used to calculate the amplification factor. Human GADPH and hsa-miR-24c-3p were used as housekeeping. The standard curve was constructed by 5-fold serial dilutions of cDNA from 1 µg.

Mouse models

Xenograft *in vivo* assays

All *in vivo* experiments were performed in female NOD scid gamma mice (NSG, NOD.Cg-Prkdc^{scid} Il2rg^{tm1Wjl}/SzJ) purchased from Charles River (Barcelona, Spain). Animal welfare and experimental procedures were carried out in accordance with institutional (Research Ethics Committee of the University of

Granada, Spain) and international standards (European Communities Council directive 86/609). All animals (n= 6 per group) were maintained in a microventilated cage system with a 12h light/dark cycle, and they were manipulated in a laminar air-flow cabinet to keep on the specific pathogen-free conditions. For the induction of tumors, mice were anesthetized by inhalation of isofluorane, inoculated with $5x10^3$ or $1.5x10^4$ MDA-MB-231 cells by subcutaneously injection into the flank regions. Tumor growth was assessed twice weekly using a digital caliper and the tumor volume was calculated by the formula $V=(length)^2 \times width \times \pi/6$. The end-point of the experiment was at day 81, and remain mice that were still alive were euthanized by cervical dislocation.

Experimental lung metastasis *in vivo* assay

2.5×10^5 MDA-MB-231 L2T cells were injected into the tail vein of 4-6 weeks old female NSG mice (n=6 per group). Bioluminescence was monitored by IVIS at day 0 and weekly by injecting intraperitoneally 150 mg/kg of D-Luciferine (Thermo Fisher) as previously described [1]. After 4 weeks, mice were euthanized and lungs were excised, photographed for td-Tomato expression and assayed for luciferase activity washing lungs with 150 µg/mL of D-luciferine diluted in PBS. Excised lungs were also used for Hematoxylin and Eosin staining and confocal microscopy analysis.

Histological analysis

Lungs were immersed in 4% paraformaldehyde in 0.1 M PBS for 4h at 4°C, washed in 0.1M PBS and embedded in paraffin in an automatic tissue

processor (TP1020, Leica, Germany). The paraffin blocks were cut into 4mm sections for staining. Sections were deparaffinized with xylene and hydrated with decreasing alcohol concentrations (absolute to 70%), and stained with hematoxylin-eosin. Later, sections were dehydrated with increasing alcohol concentrations (95% to absolute), were cleared with xylene and mounted with mounting medium. Observation under light microscopy and digital image acquisition was carried out with an inverted microscope (Nikon H550s).

Immunohistochemical analysis

Lungs were immersed in 10% formaline at room temperature overnight, washed in 0.1M PBS, and preserved in 30% sucrose in PBS for 24h. Then, the material were soaked in OCT compound (Sakura Finitek Europe B.V., Netherlands), frozen in liquid nitrogen and blocks were stored at -40°C until use. The OCT blocks were cut into 8 mm sections and collected on SuperFrost slides (Menzel-Glasser, Germany). The sections were hydrated with PBS and mounted with mounting medium with DAPI. Observation under fluorescent microscopy and digital image acquisition was carried out with a confocal microscope (Nikon A1).

Supplementary References

1. Zaho, D. *et al.* VEGF drives cancer-initiating stem cells through VEGFR-2/Stat3 signaling to upregulate Myc and Sox2. *Oncogene* **34(24)**, 3107-3119.

Supplementary figure legends

Supplementary Figure S1. Dot-plot image and percent of $ALDH^+$ **(A)** and SP **(B)** cells of TR_1, TP, TS_1 and TS_2 subpopulations of MCF7, MDA-MB-231, HT-29 and T84. **(C)** CD326 change in TR_1, TP, TS_1 and TS_2 of HT-29. **(D)** Proportion of $CD133^+$ cells for the different subpopulations of T84. Data shown as mean ± SD (**P<0.01;*P<0.05).

Supplementary Figure S2. Rate of ALDH+ cells for different subpopulations of MCF7 obtained by the method describe in Fig 1B. Data shown as mean ± SD (**P<0.01;*P<0.05).

Supplementary Figure S3. Flow cytometry for the expression of cytokertin 18 and cytokeratin 20, for breast and colon cancer cell lines respectively, in the TS_1 subpopulation after 7 days differentiation in media containing 10% FBS in adherence and compared with the expression in the TP.

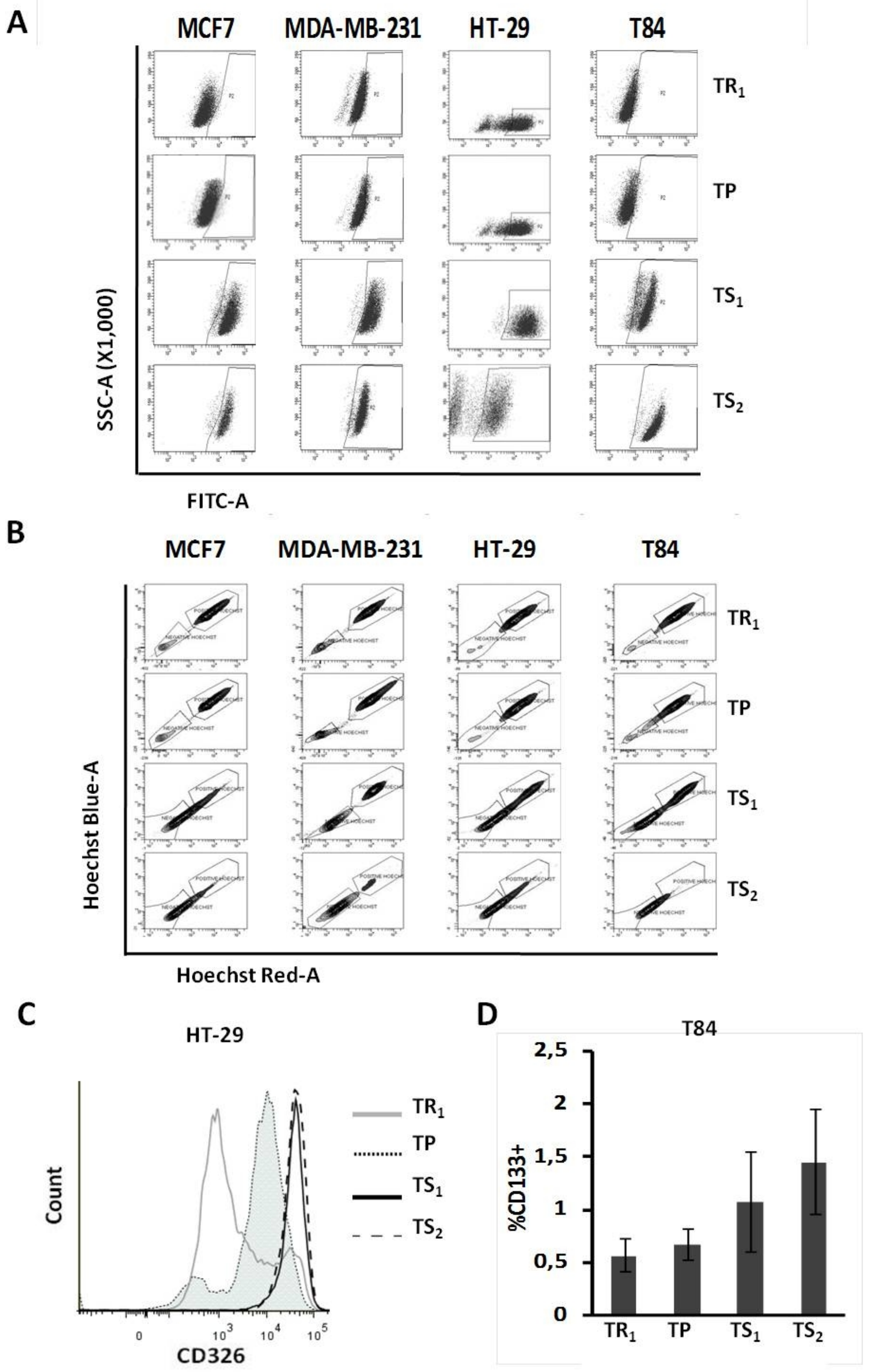
A
MCF7
MDA-MB-231
HT-29
T84
TR1
TP
TS1
TS2
SSC-A (X1,000)
FITC-A
B
MCF7
MDA-MB-231
HT-29
T84
TR1
TP
TS1
TS2
Hoechst Blue-A
Hoechst Red-A
C
HT-29
Count
CD326
TR1
TP
TS1
TS2
D
T84
%CD133+
2,5
2
1,5
1
0,5
0
TR1
TP
TS1
TS2

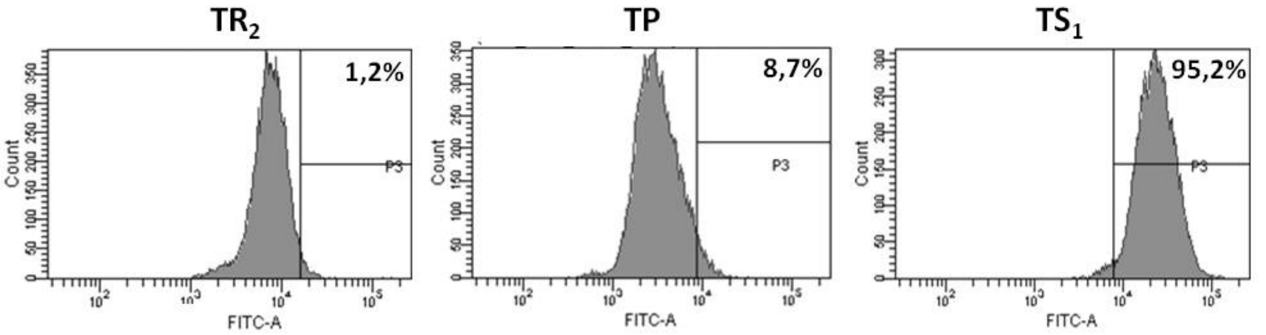

TR$_2$
TP
TS$_1$
1,2%
8,7%
95,2%
P3
Count
FITC-A

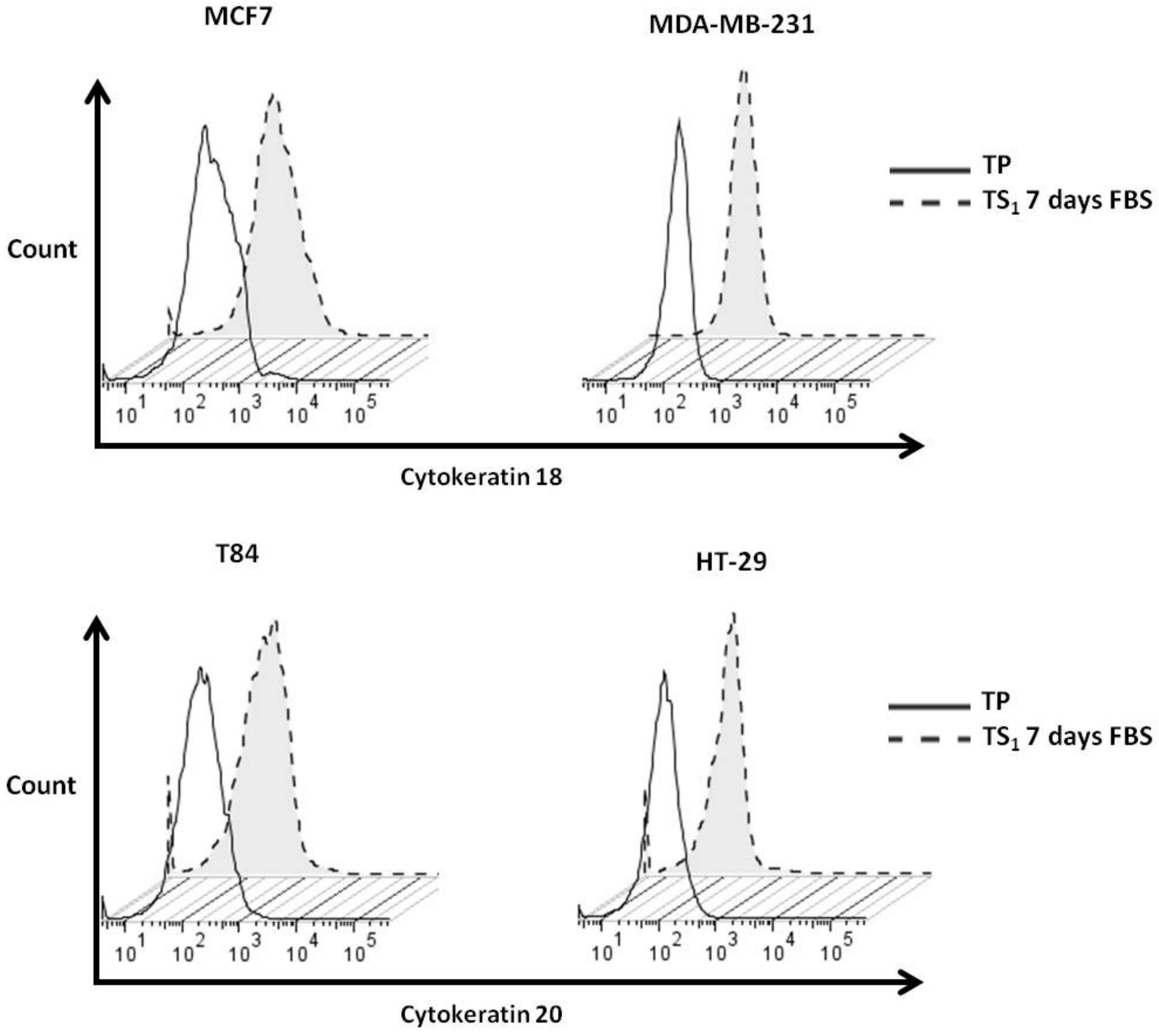

MCF7
MDA-MB-231
TP
TS1 7 days FBS
Count
10 1
10 2
10 3
10 4
10 5
Cytokeratin 18
T84
HT-29
TP
TS1 7 days FBS
Count
Cytokeratin 20
